# Prolonged Lecture Terms

**Published:** 1850-10

**Authors:** 


PROLONGED LECTURE TERMS.

We have repeatedly expressed our views in opposition to the lengthening of the lecture terms in medical schools, beyond four months, and the opposition is based upon the belief that there may be such a thing as too much lecturing. That more can be said in six months than in four is undoubtedly true; but that more can be said profitably to students in the long term, than in that which custom and experience have prescribed we are not prepared to admit. The principle upon which this opinion is based is a very obvious one. Sir Walter Scott bears testimony to a kindred one, when he says that one reason why boys of limited means of acquiring knowledge make themselves the greatest masters of it, is that they are restricted to a few books, upon which they concentrate their powers, instead of dividing their energies over a multiplicity of means as boys with better resources do. And thus it is with medical teachers and pupils, as a general rule—the four months' term makes consciseness on the part of the teachers, and concentration on the part of the pupils, essential elements of a course, and much would be lost on both sides by the six mouths term. A recent lecture by Mr. Guthrie, at the Westminster Hospital clearly sustains this view. That gentleman has had ample experience in the subject of which he speaks. He is a veteran in the profession; from an early period of his life he has been engaged in arduous duties, and enjoyed, under Wellington, an ample field for observation. He became a member of the Council of the Royal College of Surgeons in 1824, of which body he has twice been President, and, during five years, filled the office of Professor of Anatomy and Surgery in that College. He has lectured on surgery, in London, nearly forty years. This venerable man, ripe with the richest experience, and full of the amplest knowledge, in a recent lecture at the Westminster Hospital, "on some of the more important points in surgery," says: "I think, gentlemen, you have enough for one day, and more particularly if you will permit me to say, without intending offence to any one, whether teacher or student, that few, very few, of the candidates for the diploma of the College of Surgeons, who appear before me, as one of their examiners, know scarcely anything of what I have been explaining to you. Indeed, if you will further permit me, I will hint to you, with the greatest possible delicacy, that very few gentlemen of the present day appear to pay that attention to their studies, they did formerly, and the consequence is, that there are many not so well informed as students were twenty years ago, although three years of hospital and anatomical study are now required, instead of two."

When students concentrated their energies upon the course of two years, they were economic of time, but when they had three years' probation to undergo, they were prodigal of time, and economic of labor, and the result is as Mr. Guthrie details it. We are tempted to believe that a similar result would follow the lengthening of the lecture terms to six months. B.

